# Monoaminergic modulation of photoreception in ascidian: evidence for a proto-hypothalamo-retinal territory

**DOI:** 10.1186/1741-7007-10-45

**Published:** 2012-05-29

**Authors:** Florian Razy-Krajka, Euan R Brown, Takeo Horie, Jacques Callebert, Yasunori Sasakura, Jean-Stéphane Joly, Takehiro G Kusakabe, Philippe Vernier

**Affiliations:** 1 Neurobiology and Development, UPR3294, Institut de Neurobiologie Alfred Fessard, Centre National de la Recherche Scientifique, Gif-sur-Yvette, 91190, France; 2 Department of Animal Physiology and Evolution, Stazione Zoologica Anton Dohrn, Naples, 80121, Italy; 3 Shimoda Marine Research Center, University of Tsukuba, 5-10-1 Shimoda, Shizuoka 415-0025, Japan; 4 Laboratoire de biochimie, Hôpital Lariboisière, Paris, 75010, France; 5 Department of Biology, Faculty of Science and Engineering, Konan University, Kobe 658-8501, Japan; 6 Japan Science and Technology Agency, PREST, 4-1-8 Honcho, Kawaguchi, Saitama 332-0012, Japan; 7 New York University Center for Developmental Genetics, Department of Biology, New York University, 1009 Silver Center, 100 Washington Square East, New York, NY 10003-6688, USA; 8 Institute of Biological Chemistry, Biophysics and Bioengineering, School of Engineering and Physical Sciences, Heriot-Watt University, Edinburgh EH14 4AS, UK

## Abstract

**Background:**

The retina of craniates/vertebrates has been proposed to derive from a photoreceptor prosencephalic territory in ancestral chordates, but the evolutionary origin of the different cell types making the retina is disputed. Except for photoreceptors, the existence of homologs of retinal cells remains uncertain outside vertebrates.

**Methods:**

The expression of genes expressed in the sensory vesicle of the ascidian *Ciona intestinalis *including those encoding components of the monoaminergic neurotransmission systems, was analyzed by in situ hybridization or *in vivo *transfection of the corresponding regulatory elements driving fluorescent reporters. Modulation of photic responses by monoamines was studied by electrophysiology combined with pharmacological treatments.

**Results:**

We show that many molecular characteristics of dopamine-synthesizing cells located in the vicinity of photoreceptors in the sensory vesicle of the ascidian *Ciona intestinalis *are similar to those of amacrine dopamine cells of the vertebrate retina. The ascidian dopamine cells share with vertebrate amacrine cells the expression of the key-transcription factor Ptf1a, as well as that of dopamine-synthesizing enzymes. Surprisingly, the ascidian dopamine cells accumulate serotonin via a functional serotonin transporter, as some amacrine cells also do. Moreover, dopamine cells located in the vicinity of the photoreceptors modulate the light-off induced swimming behavior of ascidian larvae by acting on alpha2-like receptors, instead of dopamine receptors, supporting a role in the modulation of the photic response. These cells are located in a territory of the ascidian sensory vesicle expressing genes found both in the retina and the hypothalamus of vertebrates (*six3/6, Rx, meis, pax6*, visual cycle proteins).

**Conclusion:**

We propose that the dopamine cells of the ascidian larva derive from an ancestral multifunctional cell population located in the periventricular, photoreceptive field of the anterior neural tube of chordates, which also gives rise to both anterior hypothalamus and the retina in craniates/vertebrates. It also shows that the existence of multiple cell types associated with photic responses predates the formation of the vertebrate retina.

## Background

The vertebrate retina is made of multiple cell types, including photoreceptors, bipolar, amacrine, horizontal and ganglion cells and glia [1]. Although photoreceptors are widespread in animals, the other retinal cell types have no obvious counterparts in non-vertebrate species, and as a result, the question of their evolutionary origin is left uncertain [2-4]. Based on molecular fingerprints (mostly the expression of transcription factors and components of the phototransduction machinery), two categories of photoreceptor cells (PRCs), ciliary and rhabdomeric PRCs, once thought to be respectively specific to vertebrates and protostomes, were proposed to be sister cell types instead, and co-exist in most bilaterian species [5]. In addition, strong evidence supports the view that ganglion cells of the vertebrate retina could be derived from ancestral rhabdomeric PRCs, whereas rods and cone PRCs may originate from ancestral ciliary PRCs [2-4]. Thus, rods, cones and ganglion cells would be the earliest cellular components of the retina of ancient craniates (craniates include vertebrates plus the hagfishes, a group of marine species which may lack true vertebra but have most characters in common with vertebrates [6,7]). Supporting this contention, the adult hagfish retina is only made of rods and cones connected to ganglion cells [8,9]. Since the retina of lampreys (proposed to be the sister group of hagfish within the cyclostomes [10]) exhibits the whole complement of vertebrate retinal cell types [11], the diversification of retinal cell types should have occurred after the divergence of the hagfish ancestor from other jawless vertebrates, contradicting the proposed monophyly of cyclostomes. The alternative hypothesis would be that the multiple retinal cell types were generated before the emergence of craniates from ancestral chordates and that the hagfishes, which live in the dark, have secondarily lost several of the retinal cell types. Indeed, other work recently suggested that the bipolar cells of the vertebrate retina could be evolutionarily related to the transmedullary neurons of the eye of *Drosophila *and other protostomes, whereas vertebrate ganglion cells may be homologous to the projection neurons of the lobula complex of *Drosophila *and other insects [4]. This hypothesis sets the diversification of retinal cell types much earlier in bilaterian evolution. Overall, the question of the origin of the different cell types of the vertebrate retina would benefit from studies in species having diverged closer to the emergence of craniates/vertebrates.

In this respect, the ascidian tunicates, such as *Ciona intestinalis*, are of interest since they belong to the sister group of craniates [12,13], and their larval central nervous system shares a regionalization pattern with vertebrate brains [14,15]. Three distinct groups of PRCs have been depicted within the sensory vesicle of the ascidian larva [16]. Two of the ascidian PRC groups are associated with the pigmented ocellus, while the third one corresponds to a non-pigmented ocellus. However, the question of the existence of homologues of retinal cell types of vertebrates other than PRCs in the sensory vesicle of ascidians had not been addressed so far.

In particular, amacrine cells of the vertebrate retina make a class of auxiliary cells expressing various neurotransmitters, such as GABA, glycine, serotonin (5-HT), but also dopamine (DA) [1]. They are conserved among craniates [17], except hagfish [8]. In ascidians, a single group of dopamine-synthesizing cells exists, which is located on the left side of the postero-ventral wall of the sensory vesicle, adjacent to the third group of photoreceptors [18]. We investigated here whether they could share similarities with dopamine amacrine cells of the vertebrate retina. We found that many molecular and functional characteristics of amacrine cells are also present in the DA cells of the ascidian sensory vesicle, rendering plausible the idea that the two cell types share a common evolutionary origin.

## Results

### Similarities between dopamine cells of the sensory vesicle of *Ciona *and amacrine cells of the vertebrate retina

The single population of dopamine-synthesizing cells in the sensory vesicle of *Ciona intestinalis *was previously described to co-express the transcription factor-encoding genes *CiMeis *and *CiSix3/6 *with transcripts encoding tyrosine hydroxylase, the limiting enzyme of catecholamine biosynthesis (*CiTH*) [18]. Since the vertebrate hypothalamus also comprises several populations of DA-synthesizing cells, this finding was taken as evidence for the presence of the *CiTH*-positive cells in a protohypothalamic domain [19]. However, since the vertebrate counterparts of *CiMeis *and *CiSix3/6 *are both found in the retina [20-22], and since the DA cells of the sensory vesicle of ascidian larvae are located close to photoreceptors, we wanted to test the hypothesis that they may also be homologous to vertebrate amacrine cells.

**Figure 1:**
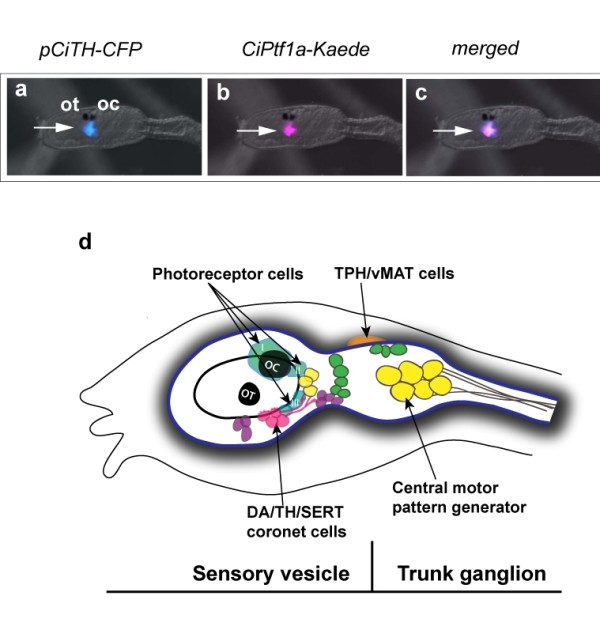
**Expression of *CiPtf1a *in dopamine cells of the sensory vesicle of *C. intestinalis *larva**. Dorsal views of heads of *C. intestinalis* larvæ expressing CFP in TH cells (pSP-Ci-TH-CFP) **(a)**, as well as the Kaede reporter under the control of the *CiPtf1a* promoter (pSP-Ci-Ptf1a Kaede) **(b)** in the sensory vesicle of ascidian larva; merged image **(c)**. Ot : otolith and Oc : ocellus. (anterior to the left, scale bar, 50 µm). Ot, otolith and Oc, ocellus. (scale bar, 50 μm). **(c) **Schematic drawing of a lateral view of the head and neck of an ascidian larva (the tail is not shown). The head CNS is shaded and divided into sensory vesicle the trunk ganglion. The neuronal populations expressing identified neurotransmitters are grossly shown. The DA coronet cells (pink), also expressing SERT and Ptf1a, are in close relationships with the population III of photoreceptors and one population of cholinergic neurons (yellow). They send processes toward glutamatergic (purple) and GABAergic (green) neurons. The TPH/vMAT expressing cells, which are localized dorso-laterally to the CNS are shown only on the right side. The central motor pattern generator and mostly the cholinergic output neurons are shown.

Based on the expression pattern of transcription factors accessible in the *Ciona *ghost database http://ghost.zool.kyoto-u.ac.jp[23] and the Aniseed database http://aniseed-ibdm.univ-mrs.fr[24], we identified *CiPtf1a *as a gene of particular interest, as its vertebrate homologue is specifically expressed in amacrine cells, many of which synthesize DA in addition to GABA [25,26]. Identification of the *CiPtf1a *transcript by *in situ *hybridization at larval stage suggested it was localized in the same cells as *TH*, which was confirmed by the co-expression of fluorescent reporters driven by the promoters of genes encoding *CiTH *(p*CiTH-CFP*) and *CiPtf1a *(*CiPtf1a-Kaede*) (Figure 1a-c). The *TH*-*Ptf1A *co-expression brings a significant evidence of the similarities that exist between DA cells of the ascidian sensory vesicle and amacrine cells of the vertebrate retina.

**Figure 2:**
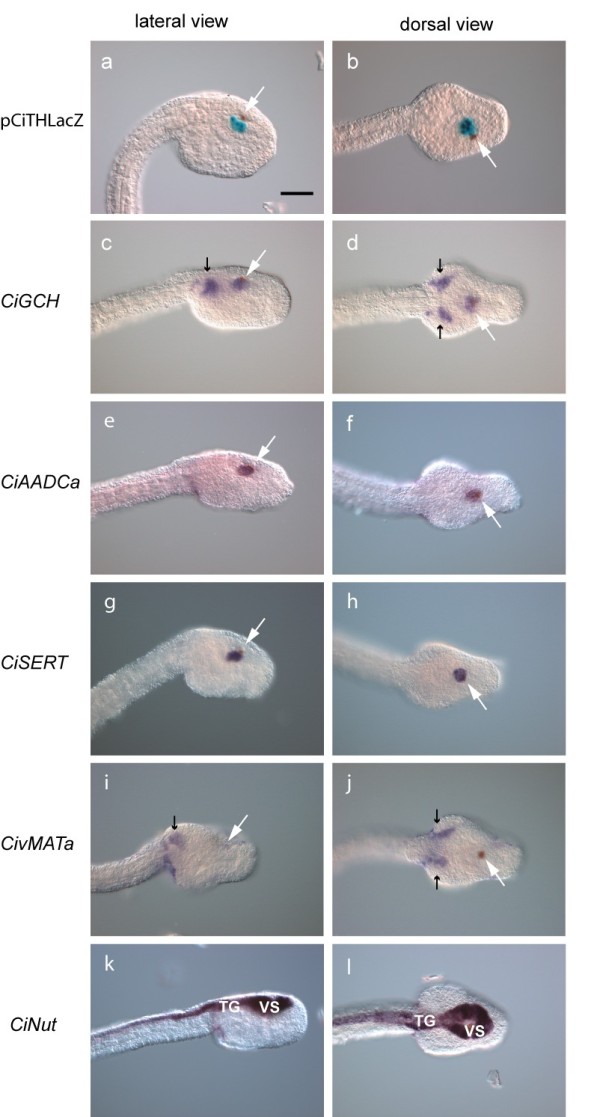
**Expression of monoaminergic markers in *C. intestinalis *embryos**. Lateral (**a, c, e, g, i, k**, anterior to the right) and dorsal views (**b, d, f, h, j, l**, anterior to the right) of late-tail-bud-stage embryos. (a, b) β-galactosidase activity driven by the *CiTH *cis-regulatory sequence (pCiTH-LacZ) (dopamine cells) is detected in the ventral sensory vesicle. (c-l) *In situ *hybridization of the indicated probes. White arrows point to the pigmented otolith. (c, d) *CiGch *is expressed in two domains, one corresponding to the *CiTH *expressing cell population within the sensory vesicle and the other to the *CiTph *territory flanking the trunk ganglion (small black arrows). *CiAadc-a *(e, f) and *CiSERT *(g, h) are expressed in the *CiTH *domain, while *CivMat-a *(i, j) is expressed at the same location as *CiTph*, outside of the nervous system (small black arrows). To accurately localize these transcripts, we used *CiNut *(k, l) as a pan-neural marker expressed in the CNS including the sensory vesicle (SV) and the trunk ganglion (TG). Sense probes gave no signal (data not shown). Scale bar = 20 μm.

To further substantiate the phenotype of these dopaminergic cells [18], we identified the whole complement of genes coding for monoaminergic markers in the published genomes of *Ciona intestinalis *[27] and *Ciona savignyi *[28] by comparison with vertebrate sequences (see list in Additional file 1, Table I). As analyzed by *in situ *hybridization in late tail-bud embryos, monoaminergic markers were located in two regions: (i) the ventral part of the posterior sensory vesicle corresponding to the *CiTH *population previously described [18], and (ii) a bilateral cell cluster located posteriorly, outside the sensory vesicle, as revealed by the *CiNut *hybridization signal, a pan-neuronal marker (Figure 2a-j). The second cluster probably corresponds to cells expressing tryptophan hydroxylase (*CiTPH*), the key enzyme of serotonin synthesis, which flanks the trunk ganglion [29], formerly named visceral ganglion [14]. Transcripts of GTP cyclohydrolase I (*CiGCH*), the key enzyme in the synthesis of tetrahydrobiopterin, an essential cofactor of both TH and TPH, was the only marker present at both sites (Figure 2c, d). Two genes encoding vesicular monoamine transporter (*CiVMAT*) and two genes encoding aromatic amino acid decarboxylase (*CiAADC*) were found in the *Ciona *genomes, as the result of a specific duplication in the ascidian lineage. One of the *vMAT *genes is expressed specifically in the *TPH *peripheral domain while the other one did not show any signal (Figure 2i, j). In contrast, no signal for any of the *AADC *genes was detected in the *CiTPH*-expressing territory, whereas the second *AADC *is expressed at the same location as the *TH *cells (Figure 2e, f). No sequence related to the plasma membrane DA or noradrenaline transporters (DAT and NET) exists in the *Ciona *genomes, probably a secondary loss in the ascidian lineage, whereas a serotonin transporter-like sequence (CiSERT) was present, as previously reported [30]. Most surprisingly, *CiSERT *transcript appeared to be expressed in the *TH *but not in the *TPH *domains of the tail-bud embryos and larvae (Figure 2g, h). Finally, these cells displayed bulbous protrusions (coronets) into the cavity of the sensory vesicle (see Additional file 1 Figure S2 h-j), as previously described [18].

**Figure 3:**
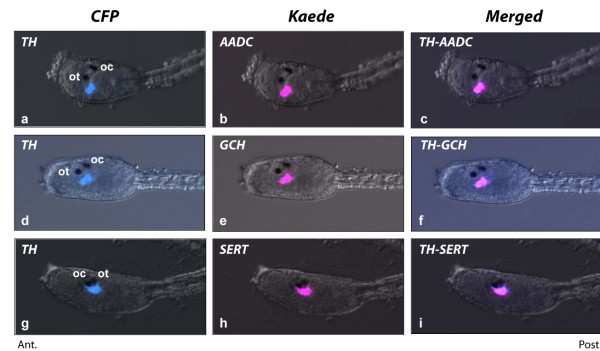
**Promoter-driven co-expression of TH, AADC and SERT in the same cells of sensory vesicle**. Views of heads of *C. intestinalis *larvae co-expressing CFP in TH cells (*pSP-Ci-TH-CFP*, **a, d, g**) and Kaede under the control of the AADC promoter (*pSP-Ci-AADC-Kaede*, b) GCH promoter (*pSP-Ci-GCH-Kaede*, **e**) or the SERT promoter (*pSP-Ci-SERT-Kaede*, **h**). Merged images (**c, f, i**) demonstrate the co-localization of *TH, AADC, GCH *and *SERT*-driven fluorescent reporters in the DA cells of the *Ciona *sensory vesicle. Ot, otolith and Oc, ocellus. Ant., anterior to the left, Post, posterior to the right for all the images.

The *TH*-expressing cells were shown to contain DA [18] and, as such, they should co-express AADC, the second enzyme in the DA synthesizing pathway. Indeed, the *AADC *promoter drove the expression of the Kaede reporter protein in the same cells that also displayed the *TH*-driven CFP fluorescence (Figure 3a-c). Those cells also co-localize the fluorescent reporter under the control of the *GCH *(Figure 3d-f) and *SERT *promoters (Figure 3g-i), and are thus characterized as DA-synthesizing cells exhibiting a SERT-like transporter.

The presence of *SERT *in DA cells of the sensory vesicle prompted us to look for the presence of 5-HT-immunoreactivity in the TH-expressing cells of *Ciona *embryos, larvae and juveniles. No 5-HT staining was observed in embryos and larvae (see Additional file 1, Figure S1a, b), although 5-HT-positive cells were found at several locations in the juveniles (see Additional file 1, Figure S1d-f). This is probably due to the poor sensitivity of 5-HT immunolabelling, since 5-HT could be detected by fluorometry after HPLC even at the earliest stages. The levels of 5-HT decreased from egg (3.5 ± 2.9 fmoles/individual) to larva (1.8 ± 1.5 fmoles/individual) before increasing again during the post-metamorphic stages (see Additional file 1, Figure S1g), suggesting that the origin of 5-HT is maternal throughout pre-metamorphic life.

Then, larvae treated with exogenous 5-HT (see Methods) were immunostained to test for 5-HT uptake capacity (as is the case in amacrine cells of the vertebrate retina [31]). In this condition, specific 5-HT immunolabelling was easily detected in the *CiTH-*expressing cells of the ventral sensory vesicle (co-localization with the Venus fluorescent protein driven by the *CiTH *promoter (see Additional file 1, Figure S2a, b). 5-HT uptake was partially inhibited by a pre-treatment with Fluoxetine, a selective SERT blocker (see Additional file 1, Figure S2c). Similarly, 5-HT accumulated in a few cells of the ventral sensory vesicle of another ascidian species, *Phallusia mammillata*, indicating that this is not a peculiarity of *C. intestinalis *(see Additional file 1, Figure S2d). It is thus very likely that 5-HT could be stored together with DA in the *CiTH-*expressing cells of the sensory vesicle, a property shared by some vertebrate amacrine cells [31].

### Investigating the role of DA/5HT neurotransmission on motor control in ascidian larvae

The possible similarities between DA-synthesizing/5HT-accumulating cells of the ascidian sensory vesicle with DA amacrine cells of the vertebrate retina prompted us to test whether these cells would be able to modulate responses to light in the ascidian larvae. This functional role was analyzed on the swimming behavior of *Ciona *larvae through the effect of Fluoxetine, a specific blocker of SERT. In ascidian larvae, swimming activity consists of three distinct patterns: oriented tail flicks, regular spontaneous swimming and photosensitive swimming. It is driven by a central pattern generator [32,33], which resides in the trunk or motor ganglion and requires only tonic drive from the sensory vesicle to operate [33,34].

In control larvae, spontaneous swimming lasts for two to three hours after hatching, before the animal becomes sessile and begins metamorphosis [35]. Electrophysiological recordings from larval tail muscle showed that control larvae exhibited regular spontaneous swimming activity (see Additional file 1, Figure S3a, b). A brief step-down in light (1 second) induced bouts of high-speed swimming in control larvae, with an after-effect lasting for around 30 to 40 seconds (see Additional file 1, Figure S3e). The light-off activity lasted only as long as the stimulus, before the internal burst frequency gradually decreased. In the presence of 10 μM Fluoxetine, the duration of this after-effect decreased with an increase in the frequency of the underlying spontaneous bursts (see Additional file 1, Figure S3e). Fluoxetine at 20 μM abolished the spontaneous bursts and the after-effects (see Additional file 1, Figure S3e) and washing failed to completely reverse the effects.

To get insight into the location of the effect of the SERT blockade within the motor network, the larvae were severed at the neck region (Figure 1d). This procedure resulted in "headless" larvae that did not swim (see Additional file 1, Figure 3f). Addition of L-glutamate to such preparations induced swimming activity very similar to the spontaneous swimming observed in intact larvae (see Additional file 1, Figure S3g), but application of 20 μM Fluoxetine did not promote obvious change in either frequency or duration of the swimming bursts (see Additional file 1, Figure S3h, i). This result suggested that the effect of SERT blockade took place in the sensory vesicle to modulate both spontaneous and shadow response swimming. Taken together, these data showed that the blockage of ciSERT by Fluoxetine is able to modulate spontaneous and light-triggered motor responses, probably through the generation of an increase in extracellular DA and 5HT.

### Monoamines modulate photic responses via α2 adrenoreceptors on target neurons in *Ciona*

**Figure 4:**
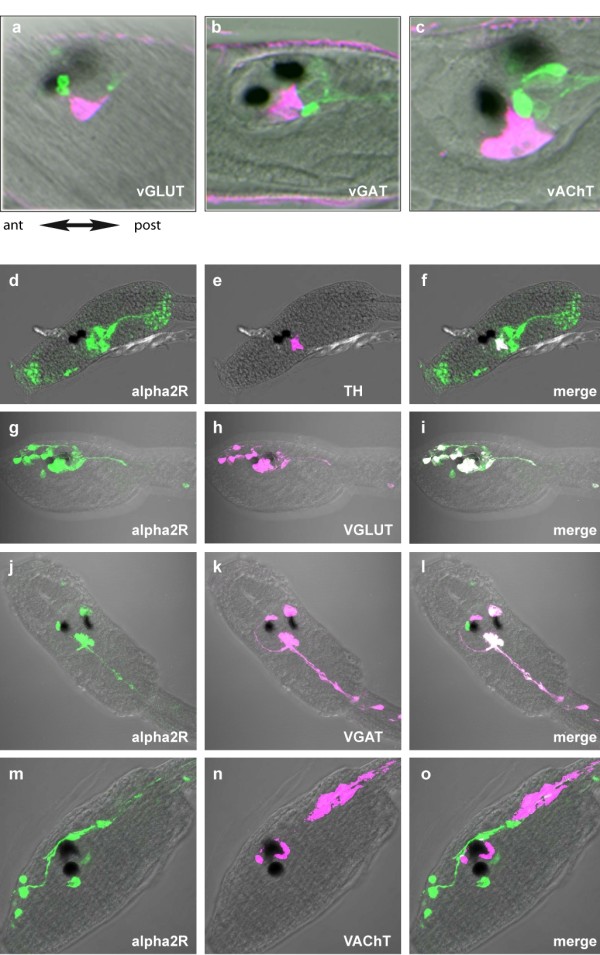
**Expression of *CiTH *and of *Ci-ADRα2a *receptor in the neuronal network of *C. intestinalis *larva**. Confocal sections of larvae co-electroporated with *pCi-TH-WGA *and constructs reporting expression of the following neurotransmitter markers: **(*a, d*) **glutamate transporter (*pCi-vGluT-eGFP*), **(*b, e*) **GABA transporter (*pCi-vGaT-eGFP*) and **(*c, f*) **acetylcholine transporter (*pCi-VAchT-eGFP*). Localizations of WGA (red) and eGFP (green) were visualized by immunofluorescent staining with an anti-WGA antibody and an anti-GFP antibody, respectively. Merged images (*a, b, c*) corresponds to merged images with Nomarski channel (*d, e, f*). A, anterior, P, posterior. (Scale bar, 25 μm in (*a, b*), 10 μm in c.). (**c-n**) Confocal sections of larvae with co-electroporated *pCi-ADRα2a-Venus *and constructs reporting expression of the following markers of neurotransmitter systems: (c, d, e) dopamine (*pCiTH-Kaede*), (f, g, h) glutamate (*pCi-vGluT-Kaede*), (i, j, k) GABA (*pCi-vGaT-Kaede*) and (l, m, n) acetylcholine (*pCi-VAchT-Kaede*). Localization of Venus (green) (c, f, i, l) Kaede (red) (d, g, j, m) were respectively visualized by immunofluorescent staining with an anti-Kaede antibody and an anti-GFP antibody. (d, h, k, n) merged images of anti-Kaede and anti-Venus staining. These are representative example of all the performed experiments (*n *= 5 to 15) depending on the couple of transgenes used.

As a possible functional parallel between amacrine and ascidian DA cells, we next examined the anatomical relationships of the ascidian DA cells with other neurotransmitter systems. The expression of fluorescent reporters driven by the promoters of genes encoding vesicular transporters for glutamate (*CivGLUT *[36]), GABA (*CivGAT *[37]) and acetylcholine (*CivACHT *[37]) was compared to that of *CiTH*. The corresponding constructs appeared to be expressed in neurons neighboring the *CiTH*-expressing cells. Specifically, DA cells made large somatic contacts with the glutamatergic, GABAergic and cholinergic neurons of the sensory vesicle (Figure 4a, b and 4c respectively), suggesting that the transmitters released by the CiTH-expressing cells could modulate the activity of target cells by non-synaptic mechanisms ("volume transmission").

We looked for the presence of monoamine receptors in the sensory vesicle of *C. intestinalis*. Surprisingly, the genome of *Ciona *does not contain any sequence resembling the bilaterian D1- or D2-like receptor gene [38]. In contrast, we identified nine sequences potentially encoding monoaminergic-like receptors, including five 5-HT-like receptors (*Ci5-HT1-a, Ci5-HT1-b, Ci5-HT2, Ci5-HT7-a and Ci5-HT7-b*) and four adrenergic-like receptors (ADR, two ADR*β*-like receptors, *CiADRβ-a *and *CiADRβ-b *and two ADR*α*2-like *CiADRα2-a *and *CiADRα2-b*; see Additional file 1, Information table II). Since D1-like and D2-like receptors are present in most protostomes and deuterostomes, they are likely to have been secondarily lost in *Ciona*. Nonetheless, several ADR receptors were shown to respond to dopamine in vertebrates [39,40], as well as in amphioxus [41] raising the possibility that in *C. intestinalis*, the target of DA signaling are the ADR-like receptors.

To test this hypothesis, we analyzed the localization of three out of four ADR-like receptors transcripts in *Ciona intestinalis *larva: *CiADRβ-a, CiADRβ-b *and *CiADRα2-a *and. Only *CiADRα2-a *transcripts were detected in the sensory vesicle and also in some peripheral neurons (Figure 4d-o, and data not shown). The cells expressing *CiADRα2-a *were more precisely identified by the distribution of the fluorescent protein driven by the corresponding 5' upstream regulatory sequences. The expression of the reporter transgenes displayed some mosaïcism from one experiment to another, but the pattern obtained in dozens of larvae in at least five independent experiments per transgene was always very similar and consistent. The p*CiADRα2-a *driven fluorescence was localized in the *CiTH *expressing cells (Figure 4d-f), where *CiADRα2-a *may act as an autoreceptor. It is also found in glutamatergic neurons of the head (Figure 4g-i), including glutamatergic PRCs, in other glutamatergic neurons of the sensory vesicle and in rostral trunk epidermal neurons, as well as in GABAergic neurons of the sensory vesicle (Figure 4j-l) and of the middle dorsal part of the tail (not shown). Conversely, p*CiADRα2-a *drove no reporter expression in cholinergic neurons (Figure 4m-o) either in GABAergic neurons of the anterior nerve cord (Figure 4j-l) or in the glutamatergic neurons of the tail. Many of the neurons expressing *CiADRα2-a *are found at a distance from the TH cells and their processes, suggesting that DA may act through volume transmission as described in the vertebrate retina [42].

**Figure 5:**
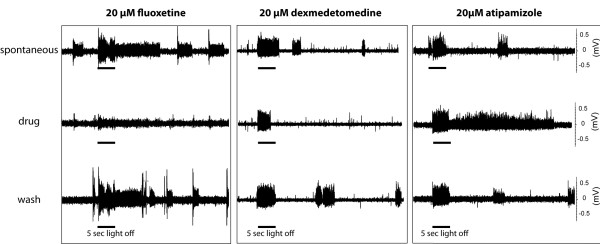
**Effect of α2 ADR agonist and antagonist on light-induced swimming response in *C. intestinalis *larva**. Muscle field potentials are recorded from the larval tail (see Methods) as an index of its contractile states. Response of *Ciona *larva to a five-second exposure to dark in control (upper trace), drug-treated (middle trace), and washed (lower trace) preparations are shown for fluoxetine (20 μM, left panel), dexmetedomidine (middle panel) and atipamizole (right panel). The blocking effect on light-off induced swimming of fluoxetine, is reproduced by the ADRα2 antagonist, whereas the ADRα2 agonist had an opposite effect, suggesting these receptors are mediating the effect of released monoamines (probably DA) on the photomotor response. These are representative example of all the experiments we have performed (*n *= 5 to 10 depending on the drug used).

A specific agonist of the ADRα2 receptor (dexmedetomidine, 20 μM) produced a decrease in spontaneous activity and a decrease in the duration of the light-off response, which terminated before the end of the light-off stimulus (Figure 5; *n *= 12). The effect was similar to that of fluoxetine (Figure 5; *n *= 20), suggesting that the neurotransmitters released from the *CiTH/SERT *cells may indeed act through *CiADRα2 *receptors. Atipamizole, an ADRα2 receptor antagonist (20 μM) had an opposing effect, increasing the duration of the spontaneous swimming bursts and the duration of the light-off effect (Figure 5; *n *= 15). These effects were very consistent and observed in all the performed experiments. All these effects disappeared upon washing. As in the case of Fluoxetine, the "headless" preparations did not show any alterations in spontaneous swimming upon treatment with dexmedetomidine or atipamizole, suggesting again that the control exerted on the photomotor response took place upstream of the central motor generator. Thus, as shown for vertebrate amacrine cells, the monoamines released by the DA cells of the sensory vesicle could modulate light responses by the activation of ADRα2 receptors on target neurons.

## Discussion

Our comprehensive examination of the molecular and functional characteristics of the DA cells of the sensory vesicle of *Ciona intestinalis *revealed unanticipated common features with the DA-producing amacrine cells of the vertebrate retina. Because DA cells of the sensory vesicle of *C. intestinalis *also share traits with the DA neurons of the vertebrate hypothalamus, a major implication of our work is that these types very likely derive from DA-synthesizing cells that were already present in a primitive proto-hypothalamo-retinal territory at the anterior neural tube of ancestral chordates. A second conclusion of these findings is that the multiple cell types found in the vertebrate retina and involved in photoreception, including PRCs, ganglion cells and amacrine cells, existed before the formation of the retina as an organ, as suggested from other studies conducted in *Drosophila *[4]. Thus, the absence of amacrine and other accessory cell types in the retina of hagfish, an animal that effectively lives in the dark, would represent a secondary loss, in agreement with the monophyly of cyclostomes [12] and with the presence of monoaminergic amacrine cells in lampreys [17].

The evidence supporting homology between vertebrate DA amacrine cells and the DA cells of the sensory vesicle of *Ciona intestinalis *are based both on shared neurochemical and molecular signatures and on functional characteristics. Beside the fact they express the whole set of molecules required to synthesize, store and release DA (TH, AADC and VMAT), the DA cells in *Ciona *display the unusual ability to take up and store serotonin (5HT). Serotonin-accumulating amacrine cells have been described in several vertebrate species [17,31,43] and, similarly, DA cells of the *C. intestinalis *larva are able to take up 5-HT via the SERT present in these cells. We postulate that this CiSERT should be able to transport DA in addition to 5HT, since no DAT (absent from the genome) is present in DA cells of the sensory vesicle, although this is an unusual property for SERT [30,44]. Supporting this contention, the blockage of CiSERT by Fluoxetine, a widely used SERT inhibitor, has a robust inhibitory effect on the swimming activity induced by shadowing, in *Ciona *larvae. This inhibitory effect is probably mediated by the action of DA on ADRα2-like receptors, since no DA receptors are present in the *Ciona *genome [38]. The action of DA through ADRα2-a receptors has also been described in the vertebrate hypothalamus [39,40]. The affinity of ADRα2-like receptors for dopamine is only about 10-fold lower than that of noradrenaline in most vertebrates [39,40], allowing them to be contingently used as DA receptors in some tissues and some species.

In vertebrates, DA amacrine cells modulate light responses of the retinal network through lateral inhibition [1,2] and the ascidian DA cells modulate the swimming behavior elicited by PRC activation. In the two cases, this effect in mediated, at least in part, by ADRα2-like receptors. In *C. intestinalis*, ADRα2-like receptors are located on DA cells, as in vertebrate amacrine cells where they act as autoreceptors [45], and also on glutamatergic photoreceptors and GABAergic interneurons, the probable targets of secreted DA. A reminiscent situation is found in vertebrates where the three paralogous ADRα2A/B/C receptor subtypes are expressed in PRCs (glutamatergic), RGCs (glutamatergic) and amacrine cells (mostly GABAergic), with some species differences [46]. A role of this DA-induced inhibition in ascidian larvae could be to promote the onset of metamorphosis, which is associated with the immobilization of the larvae and attachment to a substrate. In contrast, it was shown that blocking 5-HT receptors delayed the onset in *P. mammillata *larvae [35]. It is thus very likely that 5HT and DA have opposing effects on motor behavior in ascidians, this effect depending on the integrity of the connection between the sensory vesicle and the motor pattern generator in the trunk (see Additional file 1 Figure S3).

Incidentally, our data also highlight the large molecular flexibility and evolutionary adaptability of these DA and 5HT systems, with the loss in ascidian genomes of monoamine transporter genes except *SERT *[30], the loss of *D1-like *and *D2-like *receptors encoding genes [38]. Yet the systems remain functional by using SERT and ADRα2-like receptors for DA uptake and effect on target cells.

The similarity between DA cells of the ascidian sensory vesicle and DA amacrine cells of the vertebrate retina is reinforced by the shared expression of Ptf1a, bHLH transcription factor [25,26]. Ptf1a is mandatory for the formation of horizontal and amacrine cells, especially GABAergic amacrine cells [23]. The DA cells of *Ciona *do not contain GABA in addition to DA as in vertebrates [43], but *Ptf1a *can be expressed in all types of amacrine cells in vertebrates [26], and GABA and DA cells can be separate cell types. Other transcription factors, such as CiRx, CiSix3/6, CiPax6 and CiMeis, which contribute to retina development [20,21,47], are also localized in the anterior medial and lateral part of the sensory vesicle, where DA cells are located in *Ciona *[18,19,47,48]. Overall, these observations clearly show that DA cells are present in a photoreceptive territory in the ascidian sensory vesicle, as the amacrine cells are in the vertebrate retina.

In addition to their similarity to amacrine cells, the DA cells of the sensory vesicle of *C. intestinalis *also share traits with the CSF-contacting dopamine neurons of the vertebrate hypothalamus [18]. In *Ciona*, the ventral location of the DA cells and their protrusions (coronets) in the lumen of the sensory vesicle make them resemble the DA coronet cells of the saccus vasculosus of cartilaginous and teleost fishes, a paraventricular organ of the hypothalamus, secondarily lost in tetrapods, as well as to the dopamine-synthesizing cells of the caudal hypothalamus in teleosts [reviewed in [49]].

Based on commonalities with the hypothalamus of vertebrates, we previously hypothesized that the area of the ascidian sensory vesicle where DA cells are located was homologous as a field to the hypothalamus of the vertebrate brain [19]. Based on our present results, it seems now more likely that the sensory vesicle of ascidians on the one hand, and, on the other hand, the hypothalamus and retina together, ancestrally derive from a photoreceptive neuroepithelium folded into the anterior neural tube of ancestral chordates, as initially proposed by F.K. Studnicka in 1898, based on embryological data and a remarkable intuition [reviewed in [50]].

This hypothesis fits with the observation that deep brain photoreceptors and photoreceptive organs (retina and epiphysis) are all derived from the ventricle-lining prosencephalon [51,52]. In this respect, the ascidian PRCs would be equally related to the PRCs of the retina, the epiphysis and the hypothalamus of vertebrates. Furthermore, in the amphioxus *Branchiostoma floridae*, a non-craniate chordate which lacks a true retina, some cilliary PRCs express Pax6 [50], and these cells are part of the cerebrospinal fluid (CSF)-contacting neurons sitting in the wall of the cerebral vesicle [51,52], a region proposed to be homologous to the vertebrate prosencephalon [52,53]. In addition, DA cells of the anterior cerebral vesicle of the amphioxus are located close to these photoreceptors [54,55], a situation somewhat reminiscent of that of *Ciona*.

## Conclusion

**Figure 6:**
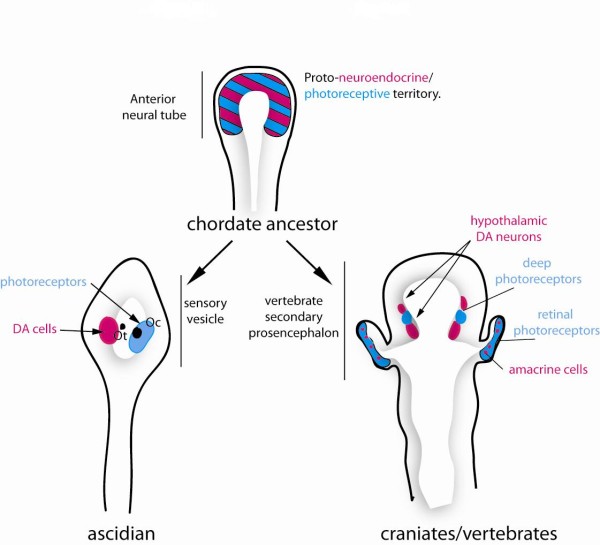
**Schematic representation of the proposed evolutionary scenario for the emergence of the retina and hypothalamus in craniates/vertebrates**. The anterior neural tube of a chordate ancestor contained a periventricular photoreceptor cells (ciliated cells, at least in part glutamatergic) intermingled with neuroendocrine cells synthesizing dopamine and neuropeptides. These cells could also be connected to the CNS. These cell types are lining the anterior neural ventricle and contact the CSF. A reminiscent but derived situation is found in modern protochordates such as ascidian (inferior left part of the drawing) where photoreceptor cells line the ventricle and are adjacent to the DA cells of the sensory vesicle, which make coronets inside the ventricle. DA cells are able to modulate the motor response to light. The situation could be very similar in the amphioxus neural tube, based on current description of the photoreceptor and DA cells. In craniates/vertebrates, the optic vesicle becomes separated from the anterior hypothalamus at the end of the neurulation process and bulged out of the neural tube to reach the lateral neuroectodermal epithelium and the lens placode, leading to new morphogenetic movements and to the inversion of the retina. The retina comprised several cell types inherited from the protochordate ancestor, including at least photoreceptor cells, pigmented epithelium and amacrine DA cells.

Based on our data and compilation of many previous studies, the following hypothesis can be put forward: The dopamine cells of the ascidian larvae derive from an ancestral multifunctional cell population located in the periventricular, photoreceptive field of the anterior neural tube of chordates, which also give rise to both anterior hypothalamus and retina in craniates/vertebrates. In vertebrates, hypothalamus and retina derive from adjacent territories of the neuroepithelium of the presumptive ventral secondary prosencephalon. Whether the hypothalamic and retinal fields are clearly separated from the origin needs to be further investigated. Then, the future retina bulges out of the secondary prosencephalon simultaneously with a movement of subduction of the hypothalamus [56]. Both retina and hypothalamus kept the potency to make photoreceptors and neurosecretory cell types, such as DA amacrine cells or DA neuronal types in the hypothalamic nuclei [52,57,58]. As a corollary, vertebrates have evolved towards subfunctionalized monoaminergic systems in the retina and hypothalamus, whereas ascidians have retained a multifunctional monoaminergic cell population (Figure 6). Involvement of DA cells of ascidian larvae in both sensory and motor modulation may represent a vestigial ancestral state. Finally, these findings also implicate that the existence of multiple cell types associated with photic responses predates the formation of the vertebrate retina.

## Materials and Methods

### Animals and embryos

*Ciona intestinalis *adults were collected in three different places: in France, from the Station de Biologie Marine de Roscoff; in Italy, from the Marine Animals Resources Service of the Stazione Zoologica A. Dohrn in the bay of Naples; in Japan, in Aioi Bay or in Murotsu Port. It is thus not possible to rule out that the animal could belong to two subspecies. However, the genes of the various proteins used in this study, the sequences of which were analyzed in the different locations were strikingly similar, and the animal morphologies also were highly similar, and this does not affect the observations and conclusion reached in this study. The animals were maintained in artificial sea water at 15 to 18°C under constant light to avoid spawning. Eggs and sperm were collected separately from dissected gonads and used in cross-fertilizations. For embryological studies, fertilized eggs were dechorionated according to [19] and raised at 13 to 18°C on 2% agarose-coated dishes in filtered artificial sea water buffered with Hepes (ASWH; under these conditions larvae hatch at around 19.5 to 20.5 hr). For post metamorphic stages, animals were transferred on non-coated dishes in ASWH and raised to the desired stage without food in order to keep them translucent. For immunohistochemistry and *in situ *hybridization, animals were fixed in PFA 4% in ASWH and 0.1% Tween, overnight or more at 4°C. After fixation, samples were progressively dehydrated in Ethanol series EtOH 25% PBS 0.1% Tween (PBSTw), EtOH 50% - PBST, EtOH 80%-H2O and eventually stored in MEOH at -20°C for several months.

### Isolation of *Ciona intestinalis *genes

Total RNA from larvae and late-tail-bud embryos of *Ciona intestinalis *were extracted with a Nucleospin extract II kit (Macherey-Nagel, Düren, Germany)). For reverse transcription, around 2 μg RNA of each developmental stage was annealed with oligo dT and hexamer random primers, followed by cDNA synthesis using M-MLV point mutant-reverse transcriptase, according to the manufacturer's protocol (Promega, Madison, Wisconsin, USA). A total of 50 to 100 ng of the resulting cDNAs were used as templates for the polymerase chain reaction (PCR), using GoTaq DNA polymerase (Promega). Specific primers used for each PCR are listed in Additional file 1, Table III. The PCR products were cloned in pCRII plasmids using TOPO TA Cloning (Invitrogen, Carlsbad, California, USA) for synthesizing the cRNA probe for *in situ *hybridization. The plasmids were subsequently sequenced and orthology of novel genes was checked by molecular phylogeny. Partial sequences of all the predicted genes were obtained (see Additional file 1, Table I). A longer fragment of *CiTph *was provided by [25] and *CiPtf1a *was retrieved from the EST library Ciona Gene Collection release 1 (clone ID cilv050i16).

### Serotonin detection and whole mount anti-serotonin immunohistochemistry

Serotonin (5-HT) was assayed as follows: *Ciona intestinalis *embryos, larvae and juveniles of the appropriate stages were counted and transferred in tubes with ASWH. They were then centrifuged at 4°C for five minutes. Supernatant was carefully removed and replaced with 400 μL a solution of a 10^-3^M chlorohydric acid solution and kept on ice before sonication, with consecutive short pulses. Then, samples were stored at -80°C before 5-HT was assayed by HPLC and fluorometric detection as previously described [59].

To test for 5-HT uptake larvae were incubated with 5-HT (Serotonin creatinine sulfate complex H7752, Sigma-Aldrich, St. Louis, Missouri, USA) (50 or 100 μM) diluted in ASWH for one hour. The uptake of 5-HT was inhibited by pre-treating larvae with Fluoxetine (50 μM) diluted in ASWH for 30 minutes before incubation with 5-HT (50 μM) in presence of Fluoxetine (50 μM) for one hour. Thereafter, larvae were quickly rinsed in ASWH and fixed in PFA 4% in ASWH - 0.1% Tween, overnight at 4°C.

Then, samples were processed for whole mount anti-5-HT immunohistochemistry essentially as described in [60]. Samples were rehydrated progressively in PBSTw and next in PBTR (PBS, Triton 0.5%, DMSO 1%), incubated one hour at RT in PBTR-NGS 1% and then incubated with a rat monoclonal anti-5-HT antibody (MAB352, Chemicon, Billerica, Massachusetts, USA) in PBTR-NGS 1% at 4°C overnight to 48 hours. Samples were then rinsed in PBTR and incubated again in PBTR-NGS 1% for one hour at room temperature and overnight incubated with the secondary antibody (biotinylated monoclonal goat anti rat antibody, Jackson ImmunoResearch Laboratories, West Grove, Pennsylvania, USA) in PBTR-NGS 1% at 4°C. After rinsing with PBSTw, the antigen-antibodies complexes were detected using the ABC peroxydase Kit (Vector Laboratories Inc., Burlingame, California, USA). Peroxydase activity was detected by using either diaminobenzidine (DAB, DiamAB Sigma) or Alexafluor568 tyramide (TSA kit, Molecular Probes, Eugene, Oregon, USA), according to the supplier's instructions. Samples were then mounted under coverslip in 50% glycerol-PBST for DAB revelation, or Vectashield with DAPI (Vector Laboratories Inc.) for fluorophore conjugated tyramides. DAB-stained specimens were observed with a Leica DMR Microscope (Leica Microsystems, Nanterre, Franceand photographed with a Nikon Digital camera DXM 1200 (Nikon, Shinjuku, Tokyo, Japan).

### Reporter gene constructs and electroporations

Different plasmid constructs were used in this study. *pCiTH-LacZ, pCi-vGluT-eGFP, pCivGAT-eGFP *and *pCi-VAchT-eGFP *are respectively the same as in [18,36,37]. The *pCiTH-WGA, pCiTH-Kaede, pCi-vGluT-Kaede, pCivGAT-Kaede *and *pCi-VachT-Kaede *were made inserting the 5' upstream corresponding sequences in front of reporter genes, either Kaede or Wheat germ agglutinin (WGA) as described in [36,37]. To generate *pSP-CiTH-Kaede, pSP-CiTH-CFP, pSP-CiPtf1a-CFP, pSP-CiAADC-Kaede, pSP-CiGCH-Kaede, pSP-CiSERT-Kaede*, the 5' upstream regions of *Ci-TH, Ci-Ptf1a, Ci-AADC, Ci-GCH *and *Ci-SERT*, were amplified by PCR using a thermostable DNA polymerase (PrimeSTAR HS DNA polymerase, Takara Bio Inc., Otsu, Shiga, Japan) and oligonucleotide primers (5'-tggatcccggtcacgggtgcctacctc-3' and 5'-gggatccatttttaaattacgtttttt-3' for *Ci-TH*, 5'-gtggatccgtatgcgtggtgtgtatgaacg-3' and 5'- ggggatccatgttatactacgttgataact-3' for *Ci-Ptf1a*, 5'-acggatccaatgacgtaataatcaactt-3' and 5'-ggggatccatttttgcagcggattctt-3' for *Ci-AADC*, 5'-agggatccggcactagattggtgtac-3' and 5'- ggggatccatattttagacctgttaatt-3' for *Ci-GCH*, 5'-ggggatcctagtttaccacgaagagtt-3' and 5'- ggggatccatattggtgattttttcaata-3' for *Ci-SERT*). The PCR products were digested with *Bam*HI and were inserted in the *Bam*HI site of *pSPKaede *and *pSPeCFP. pCiADRα2-a-Venus *was made using a Gateway cloning system (Invitrogen) as in [61] after amplification of the 5'upstream region of *CiADRα2 *with Gateway specific primers (5'-attB1-CTCGAGtggtatttcagagaactacagtttcaa-3' and 5'-attB2-CTCTAGActtccatggttaaataatcatcataca-3'). The reporter proteins were immunostained with the appropriate primary and secondary antibodies as described in [36].

### *In situ *hybridization and β-galactosidase detection

*In situ *hybridization of whole-mount specimens was carried out as described in [19]. Electroporations and X-gal staining were performed essentially as previously described in [61].

### Physiological recordings of motor behavior and modulation by monoaminergic drugs

All the drugs and compounds were purchased from Sigma unless otherwise specified. The drugs were diluted from concentrated stock solutions and added to the superfusate at the final concentrations indicated.

To test for the effects of drugs on the swimming behavior of *Ciona *larvae, video recordings of larval movements were made at five-minute intervals from individual larvae in filtered seawater with a high speed video (Fast-Cam Rabbit mini 2, Photoron Tokyo, Japan) at 400 to 500 fps mounted on a binocular microscope and stored for later analysis. In some cases, the 'head' was transected between the motor ganglion and brain vesicle (BV), disconnecting it from the motor ganglion and tail.

For electrophysiological recordings, larvae were placed either in Petri dishes containing a base of 1% agar or, in dishes coated with 1% bovine serum albumin. Both approaches prevented the larvae contacting the plastic base of the Petri dish and commencing metamorphosis. All experiments were carried out in filtered sea-water perfused over the preparation at a 8 ml/minute rate. Glass micropipettes were drawn from borosilicate glass of 1.5 mm OD on a microelectrode puller (Model P87, Sutter Instrument Co., Novato, California, USA). The tips of the electrodes were broken under microscopic control so that their internal diameter was about four-fifths the diameter of the larval tail. Coarse manipulation of the microscope stage allowed the larval tail to be brought into close contact with the tip of the electrode and negative pressure was applied to draw the larval tail into the pipette to about two-thirds of its length. Muscle action potentials were recorded differentially between the inside of the pipette and the sea-water of the bath and amplified (DAM 80, World Precision Instruments, New Haven, CT, USA) with reference to a silver chloride pellet placed in the bath. Signals were AC-coupled and passed between 0.1 Hz and 10 kHz, digitized and stored by the Digidata 1200 data acquisition system (Molecular Devices, Sunnyvale, CA, USA)) until being analyzed with Clampfit software 9.0 (Molecular Devices, Sunnyvale, CA, USA). A custom-built shutter was controlled by 5-V pulses delivered from the Digidata board allowing a step-down in the light intensity for a given time.

## Abbreviations

5-HT: serotonin; ADR: adrenergic receptor; ASWH: artificial sea water with Hepes; DA: dopamine; PRCs: photoreceptor cells; SERT: serotonin transporter; TH: tyrosine hydroxylase; Tph: tryptophane hydroxylase; vMAT: vesicular monoamine transporter.

## Competing interests

The authors declare that they have no competing interests.

## Authors' contributions

FRK conceived the study, carried out all the cloning, molecular biology experiments, phylogenetic analysis, participated in electrophysiological experiments and drafted the manuscript. EB conceived and carried out the electrophysiological and pharmacological experiments with FRK and contributed to writing the manuscript. TH made several of the transgenic constructs used to study the target of dopamine neurons in ascidian sensory vesicle, and to perform co-localization studies, supervised by YS. JC carried out the monoamine assays and interpreted the data. JSJ contributed to design and supervision of the work, and to writing the paper. TGK participated in the design of the study and contributed in analyzing most of the data. PV conceived the study, and participated in its design and coordination and wrote the manuscript. All authors read and approved the final manuscript.

## Authors' information

FRK did his PhD work in PV's team under the supervision of PV and JSJ. He is currently a post-doctoral fellow at the Department of Biology at New York University, New York, USA.

EB was group leader and head of the Department of Animal Physiology and Evolution at the Stazione Zoologica Anton Dohrn, Villa Comunale, in Naples, Italy. He is now group leader in the Institute of Biological Chemistry at Heriot-Watt University, Edinburgh, EH14 4AS, UK.

TH did his PhD under TK's supervision at Hyogo University, Japan. He is now assistant professor at the Shimoda Marine Research Center, University of Tsukuba, Japan.

YS is Associate Professor and group leader at the Shimoda Marine Research Center, University of Tsukuba, Japan. He is a world-renown specialist of the developmental biology of ascidia.

JC is full professor at the Department of Biochemistry and Molecular Biology, Hopital Lariboisière, Paris, France. His work is dedicated to the study of monoamines in human pathologies.

JSJ is group leader at the Neurobiology and Development Research Unit, CNRS, Institute of Neurobiology Alfred Fessard, Gif-sur-Yvette, France. He is a well-known specialist of the development of protochordate, especially ascidia.

TGK is professor at the Department of Biology, Konan University, Kobe, Japan and a well-known specialist of the biology of ascidia and photoreceptor systems, as well as bioinformatics.

PV is the head of the Neurobiology and Development Research Unit, CNRS and director of the Institute of Neurobiology Alfred Fessard, Gif-sur-Yvette, France, and a specialist of the evolution of nervous system in chordates.

## Supplementary Material

Additional file 1 **Data supporting the contention of the article: Monoaminergic modulation of photoreception in ascidian: Evidence for a proto-hypothalamo-retinal territory**. Two tables of the genes studied in the paper, the first one with the references of the genes encoding components of the monoamine neurotransmission pathway, the second one with the reference of the genes encoding monoamine receptors and found in the *Ciona *genome. One figure demonstrating the presence of serotonin in the ascidian larvae (Figure 1). One figure showing the uptake of serotonin in the dopamine cells of the ascidian sensory vesicle (Figure 2). One figure showing that dopamine and serotonin can modulate the light-induced swimming behaviour of the larva of *Ciona intestinalis *(Figure 3). Click here for file
