# Supplementary material for: Monoaminergic modulation of photoreception in ascidian: evidence for a proto-hypothalamo-retinal territory
Source: BMC Biol. 2012 May 29;10:45. doi: 10.1186/1741-7007-10-45 (PMC3414799; doi:10.1186/1741-7007-10-45)
Supplement: Additional file 1 — Data supporting the contention of the article: Monoaminergic modulation of photoreception in ascidian: Evidence for a proto-hypothalamo-retinal territory. Two tables of the genes studied in the paper, the first one with the references of the genes encoding components of the monoamine neurotransmission pathway, the second one with the reference of the genes encoding monoamine receptors and found in the Ciona genome. One figure demonstrating the presence of serotonin in the ascidian larvae (Figure 1). One figure showing the uptake of serotonin in the dopamine cells of the ascidian sensory vesicle (Figure 2). One figure showing that dopamine and serotonin can modulate the light-induced swimming behaviour of the larva of Ciona intestinalis (Figure 3). [file 1741-7007-10-45-S1.DOC]

**Additional file**

Data supporting the contention of the article: Monoaminergic modulation of photoreception in ascidian: Evidence for a proto-hypothalamo-retinal territory

**Florian Razy-Krajka, Euan Brown, Takeo Horie, Jacques Callebert, Yasunori Sasakura, Jean-Stéphane Joly, Takehiro Kusakabe, and Philippe Vernier**

Description:

-Two tables of the genes studied in the paper, the first one with the references of the genes encoding components of the monoamine neurotransmission pathway, the second one with the reference of the genes encoding monoamine receptors and found in the Ciona genome.

-One figure demonstrating the presence of serotonin in the ascidian larvæ (Fig.1)

-One figure showing the uptake of serotonin in the dopamine cells of the ascidian sensory vesicle (Fig.2).

- One figure showing that dopamine and serotonin can modulate the light-induced swimming behaviour of the larva of *Ciona intestinalis* (Fig.3).

| **Gene name** | **JGI name** | **Vertebrate homologues** |
| --- | --- | --- |
| ***Ci-GCH*** | ci0100148663 | GTP-Cyclohydrolase |
| ***Ci-PAH*** | ci0100145352 | Phenylalanine Hydroxylase |
| ***Ci-TH*** | ci0100143675 | Tyrosine Hydroxylase |
| ***Ci-TPH*** | ci0100149739 | Tryptophane Hydroxylase |
| ***Ci-DBH*** | ci0100154113 | Dopamine-β-Hydroxylase |
| ***Ci-SERT*** | ci0100152503 | Serotonin transporter |
| ***Ci-AADCα*** | ci0100149888 | Aromatic Amino Acid Decarboxylase  (duplication in *C. intestinalis*) |
| ***Ci-AADCβ*** | ci0100148321 |
| ***Ci-vMATα*** | ci0100140598 | Vesicular Monoamine transporter  (duplication in *C. intestinalis*) |
| ***Ci-vMATβ*** | ci0100140658 |
| ***Ci-MAO*** | ci0100154283 | Monoamine Oxidase |
| ***Ci-COMT 1*** | ci0100134953 | Catechol-O-Methyl Transferase (triplication in *Ciona*) |
| ***Ci-COMT 2*** | ci0100140047 |
| ***Ci-COMT 3*** | ci0100139394 |

Table I : List of genes encoding components of the monoamine neurotransmission pathway.

| **Gene name** | **JGI name** | **Vertebrate homologues** |
| --- | --- | --- |
| ***Ci-5HT1-a*** | ci0100137935 | 5HT1a/b/d/e/f receptors  (specific duplication in *Ciona*) |
| ***Ci-5HT1-b*** | ci0100144199 |
| ***Ci-5HT2*** | ci0100134145 | 5HT2a/b/c receptors |
| ***Ci-5HT7-a*** | ci0100140881 | 5HT7 receptor  (specific duplication in *Ciona*) |
| ***Ci-5HT7-b*** | ci0100148112 |
| ***Ci-ADREβ-a*** | ci0100130320 | ADREβ1/2 receptors  (specific duplication in *Ciona*) |
| ***Ci-ADREβ-b*** | ci0100137803 |
| ***Ci-ADREα2-a*** | ci0100132133 | ADREα2a/b/c receptors  (specific duplication in *Ciona*) |
| ***Ci-ADREα2-b*** | ci0100146328 |

Table II : List of genes encoding monoamine receptors in C*. intestinalis*.

**
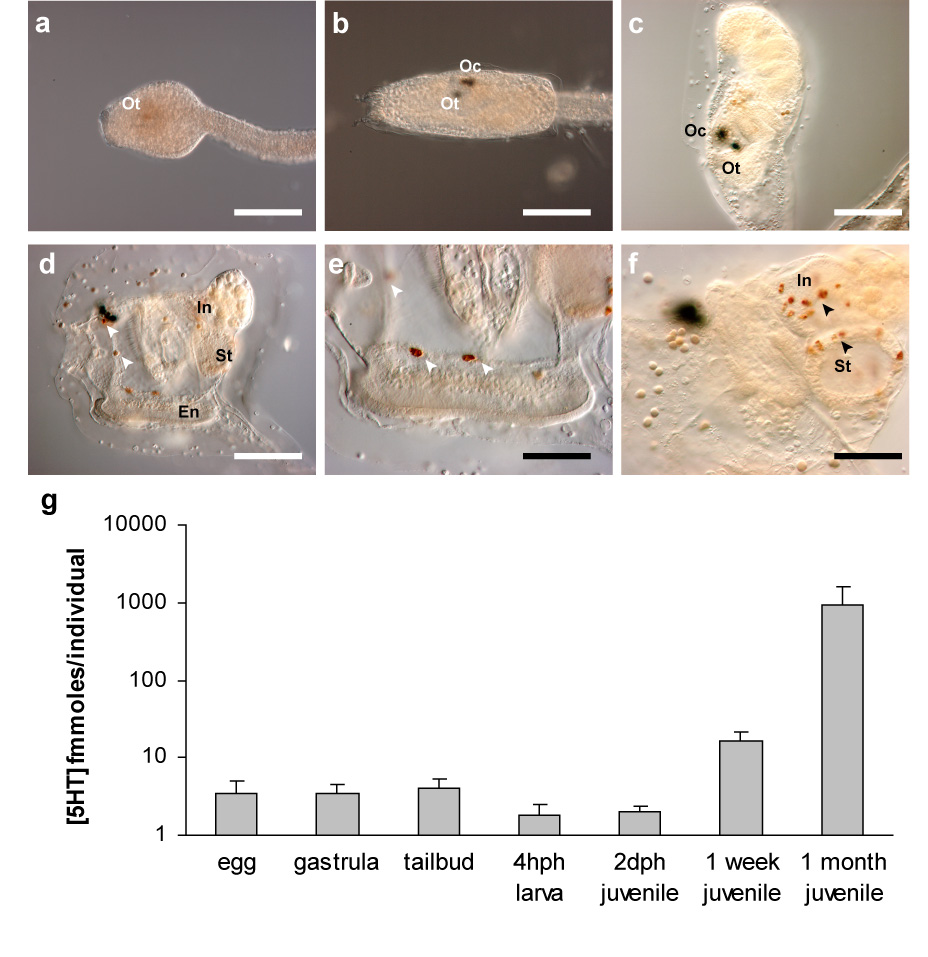
**

**Figure S1: 5-HT immunoreactivity and quantification in *Ciona intestinalis*.**

5-HT immunostaining was not detected in late-tail-bud embryo (*a*), larva (*b*) and 2 day-post-hatching juvenile (*c*). The dark spots are the otolith (Ot) and the ocellus (Oc). 5-HT immunoreactive cells were detected in 5 day-post-hatching juvenile (*d*) at several locations outside the CNS: in the peripharyngeal band beneath the neural complex (arrowheads in *d*), in the endostyle (enlarged view in *e*) and in the digestive tract tract (enlarged view in *f* with the intestine –In- and the stomach –St-; arrowheads indicate the 5-HT immunoreactive cells. (white scale bar, 100 µm in (*a*-*d*), black scale bar 50 µm in (*e*, *f*).) (*g*) 5-HT content was assayed by HPLC and electrochemical detection. at least 4 batches including 100-400 individuals, at each stage, have been assayed. 5-HT amount is 3,5±2,9 fmol 5-HT per egg and its level tends to decrease at larval stage (1,8±1,5 fmoles/individual). The concentration rapidly raises during metamorphosis, suggesting that 5-HT synthesis starts during this period.

**
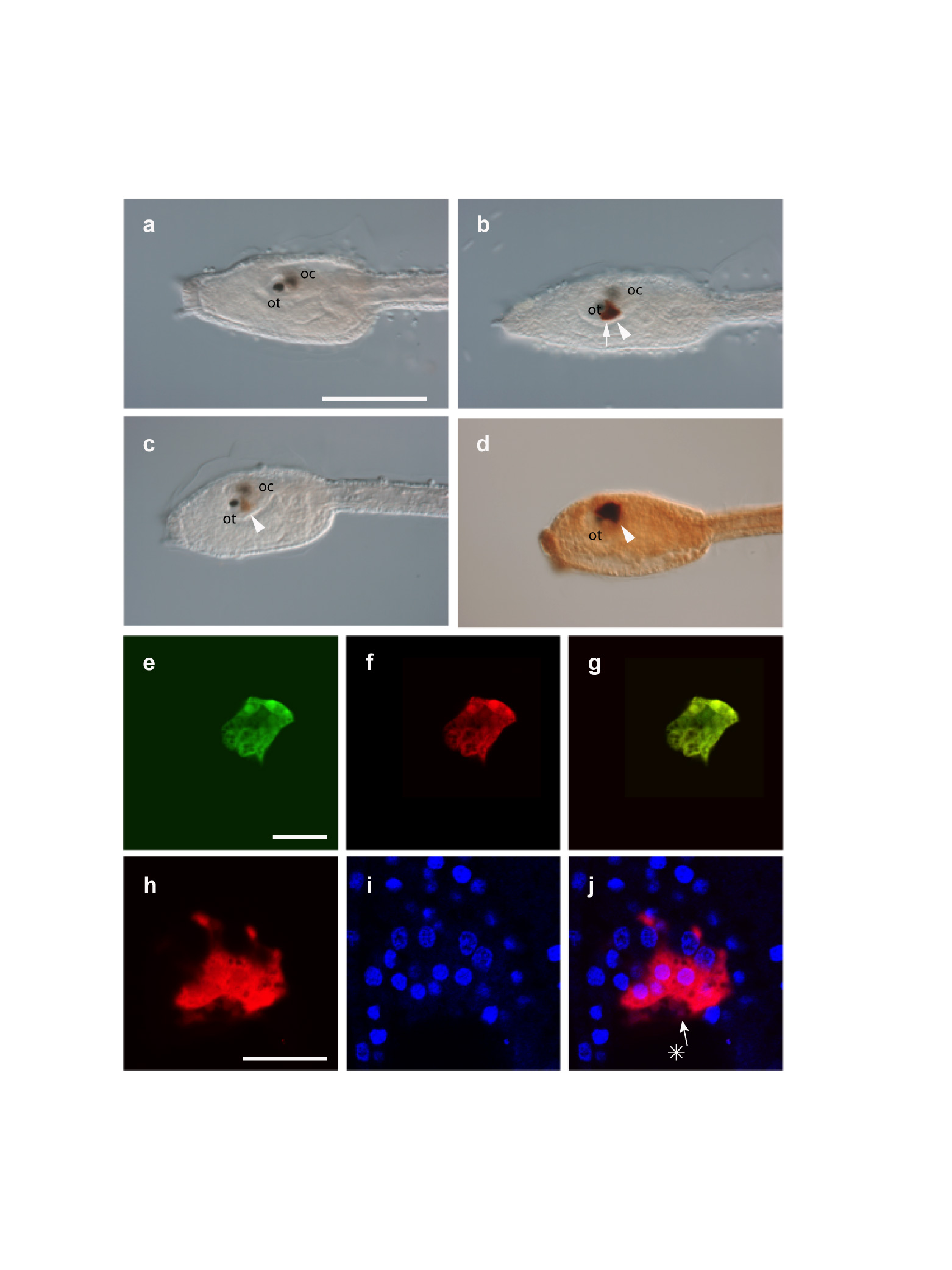
**

**Figure S2: 5-HT uptake in the *TH*-expressing cells in the larva of *Ciona intestinalis* and *Phallusia mammillata*.**

(a) Serotonin immunostaining was not detected in the control larva of *C. intestinalis* (lateral view). The dark spots are the otolith (Ot) and the ocellus (Oc). In contrast, when the larvæ were incubated with 5-HT (50µM), cells were strongly stained in the ventral sensory vesicle (arrowhead) (b). This uptake was partially inhibited in 5-HT incubated larva treated with Fluoxetine (50 µM) (c). 5-HT-accumulating cells were also observed in larvæ of *Phallusia mammillatta* incubated with 5-HT (50 µM) (d). (e-j) Confocal images of the DA cells. (e, f, g) The 5-HT accumulation takes place in the dopaminergic cells identified by pCiTH driven Venus expression (e), 5-HT immunostaining (f), merge (g). (h, i, j) The number of 5-HT-accumulating cells was counted in 5-HT treated larvae on confocal sections of 5-HT fluorescent immunostaining counterstained with DAPI. Depending on the animals the number of 5-HT accumulating cells varied between 8 and 15 (n = 7). Arrow : coronets, asterisk : cavity of the sensory vesicle. (Scale bar, 100 µm in (a-d), 10 µm in (e-j).

**
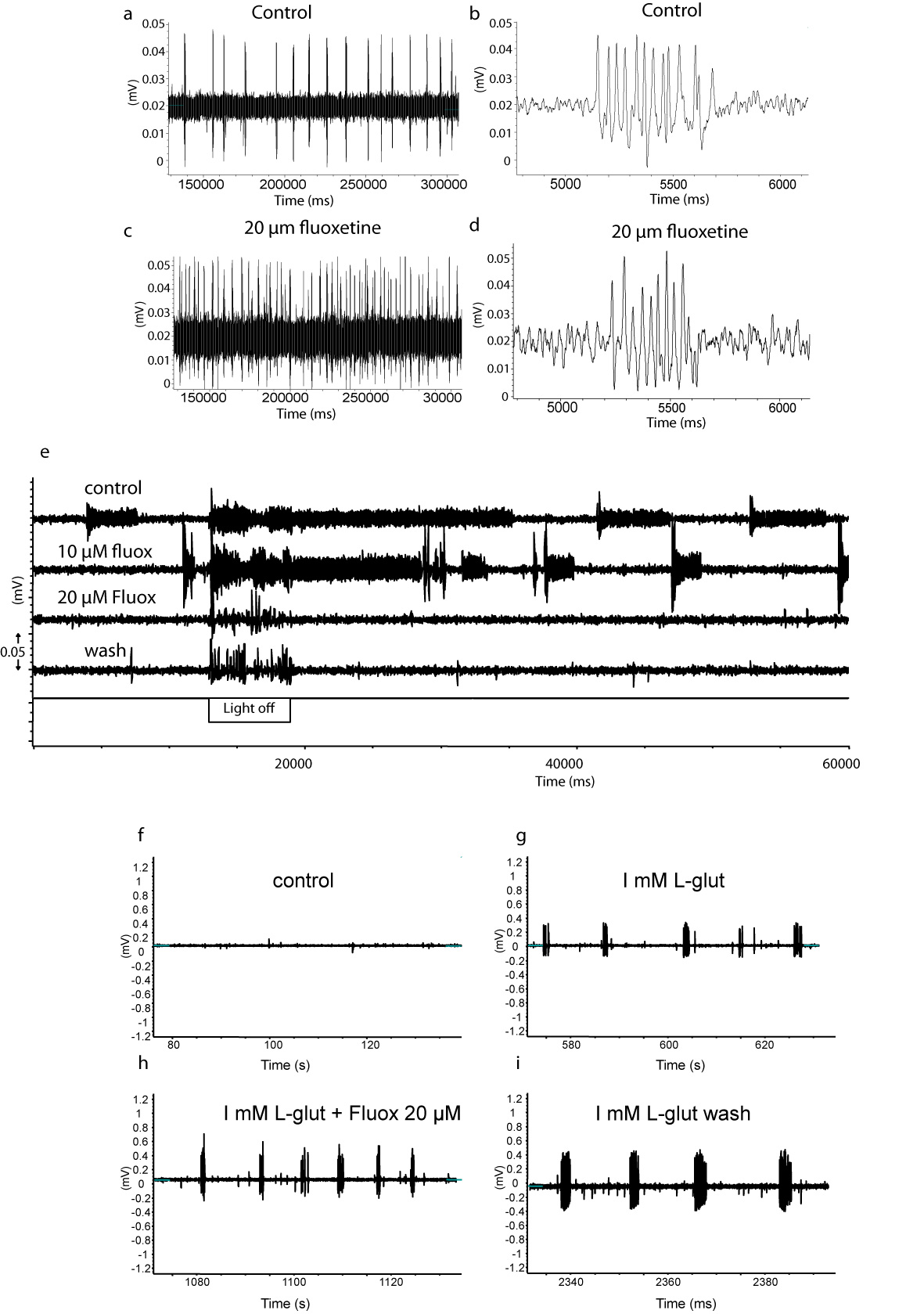
**

**Figure S3: The dopamine cells modulate the light-induced swimming behaviour of the larva of *Ciona intestinalis*.**

Muscle field potentials are recorded from the larval tail (see Methods) as an index of its contractile states. (a, b)  Example of muscle field potentials recorded for 2 min, in control conditions (a) and after exposure with 20µM floxetine in the bath (b).  (c, d)  Expanded trace of one period of field potentials shown in a and b. Note that there is no change in the frequency and amplitude of the swimming activity. (e) Response of *Ciona* larva to a one second exposure to dark in control (upper trace) and in Fluoxetine treated animals (10µM Fluox and 20µM Fluox, the two lower trances). Fluoxetine, a SERT inhibitor, completely blocked the swimming burst that occurred after shadowing the light. This response was not washable as shown on the lower trace. (f-e) Effect of Fluoxetine on glutamate-induced swimming behavior in reduced preparation of *Ciona intestinalis* larva. (f) Ciona larvæ were transected in the neck region to provide headless preparation that did not swim (Control; see text and Fig. 5 for details). (g): Glutamate (1mM) in the bath induced a swimming behaviour resembling that of control animals. (h): The addition of Fluoxetine to glutamate in the bath did not significantly change the response to glutamate. (i): After washing with medium containing again 1mM glutamate, the frequency of swimming bursts did not change, although the duration of the burst was increased, probably due to additive glutamate effect.
